# Dormant Tumor Cell Vaccination: A Mathematical Model of Immunological Dormancy in Triple-Negative Breast Cancer

**DOI:** 10.3390/cancers13020245

**Published:** 2021-01-11

**Authors:** Reza Mehdizadeh, Seyed Peyman Shariatpanahi, Bahram Goliaei, Sanam Peyvandi, Curzio Rüegg

**Affiliations:** 1 Institute of Biochemistry and Biophysics, University of Tehran, Tehran 1417614335, Iran; mehdizadeh@ut.ac.ir (R.M.); goliaei@ut.ac.ir (B.G.); 2 Laboratory of Experimental and Translational Oncology, Pathology, Department of Oncology, Microbiology and Immunology, Faculty of Sciences and Medicine, University of Fribourg, 1700 Fribourg, Switzerland; sanam.peyvandi@unil.ch

**Keywords:** breast cancer, immune-induced cancer dormancy, mathematical model, tumor heterogeneity, cancer cell vaccine, T lymphocytes, chemotherapy, type I IFN

## Abstract

**Simple Summary:**

Triple-negative breast cancer (TNBC) is the most aggressive subtype of breast cancer, particularly affecting young women. Chemotherapy is the main choice for the treatment of these patients. It has been shown that some chemotherapies induce immunogenic cell death and elicit an adaptive cytotoxic T cell immune response through the activation of the type I interferon pathway. We made an evolutionary mathematical model based on the recently reported in vivo induction of immunological tumor dormancy of a murine TNBC cell line upon in vitro treatment with chemotherapy. Our model replicates the previously obtained experimental results and predicts a prophylactic and therapeutic vaccination effect by injecting dormant cells with active type I interferon signaling, before or after challenge with the aggressive parental tumor cells, respectively. These results show the potential of a dormant tumor cell-based therapy inducing an adaptive immune response, suppressing tumor growth.

**Abstract:**

Triple-negative breast cancer (TNBC) is a molecular subtype of breast malignancy with a poor clinical prognosis. There is growing evidence that some chemotherapeutic agents induce an adaptive anti-tumor immune response. This reaction has been proposed to maintain the equilibrium phase of the immunoediting process and to control tumor growth by immunological cancer dormancy. We recently reported a model of immunological breast cancer dormancy based on the murine 4T1 TNBC model. Treatment of 4T1 cells in vitro with high-dose chemotherapy activated the type I interferon (type I IFN) signaling pathway, causing a switch from immunosuppressive to cytotoxic T lymphocyte-dependent immune response in vivo, resulting in sustained dormancy. Here, we developed a deterministic mathematical model based on the assumption that two cell subpopulations exist within the treated tumor: one population with high type I IFN signaling and immunogenicity and lower growth rate; the other population with low type I IFN signaling and immunogenicity and higher growth rate. The model reproduced cancer dormancy, elimination, and immune-escape in agreement with our previously reported experimental data. It predicted that the injection of dormant tumor cells with active type I IFN signaling results in complete growth control of the aggressive parental cancer cells injected at a later time point, but also of an already established aggressive tumor. Taken together, our results indicate that a dormant cell population can suppress the growth of an aggressive counterpart by eliciting a cytotoxic T lymphocyte-dependent immune response.

## 1. Introduction

Breast cancer is a leading cause of cancer mortality in women worldwide. Four main distinct molecular breast cancer subtypes have been defined: luminal A, luminal B, HER2-overexpressing, and triple-negative/basal-like [1,2]. Triple-negative breast cancer (TNBC) is the most aggressive subtype with the least favorable clinical outcome. It is characterized by a lack of expression of estrogen and progesterone receptors and human epidermal growth factor receptor 2 (HER-2) [3]. Targeted therapies have been introduced for luminal A/B, and HER2-amplified breast cancers, while for TNBCs, chemotherapy remains the standard form of treatment [4,5]. Anthracyclines, alkylating agents, taxanes, platinum agents, fluorouracil (5FU), and methotrexate are used as monotherapy or in combination for TNBC treatment [6,7,8,9,10].

The development of resistance to chemotherapy is a major problem in cancer treatment, including for TNBCs [11,12,13]. Many studies focus on overcoming resistance by introducing a combination of multiple cytotoxic drugs, targeted therapies, and immunotherapies [14,15,16,17,18]. High intra-tumor heterogeneity promotes the evolution of resistance that may be further modulated by active immune responses. It is hypothesized that among the different cell subpopulations present in the tumor, some are resistant to chemotherapy from the start, while others may become resistant during treatment. Based on the Darwinian principle of selection pressures in somatic evolution, the resistant cells can survive with a higher fitness after the elimination of sensitive cells, causing tumor relapse [19,20,21]. The heterogeneity of tumors may also contribute to tumor escape from the immune system by allowing the expansion of low-immunogenic subpopulations and immune-suppressing phenotypes [22,23,24,25,26].

The cytotoxic effect of some chemotherapeutic agents may be exerted through immunogenic cell death (ICD). This effect is defined by the chronic exposure of damage-associated molecular pattern antigens (DAMPs) in the tumor microenvironment (TME). ICD induces an adaptive immune response against tumor cells in an immunocompetent host [27,28]. Immune infiltrations, especially tumor-infiltrating lymphocytes (TILs), have a marked effect on the response of adjuvant and neoadjuvant chemotherapies and in determining the final clinical outcome [29,30,31]. The presence of TILs, particularly CD8^+^ T cells and tumor-infiltrating natural killer (NK) cells, correlate with an enhanced pathological complete response (PCR), a reduced risk of recurrence, and better survival in TNBC patients [32,33].

In the 1950s, Burnet and Thomas proposed the immune-surveillance hypothesis, which emphasizes the potency of the immune system to eliminate tumor cells before they are clinically detectable [34,35]. Subsequently, the cancer immunoediting theory was introduced as the evolutionary dynamics of the immune response to cancer [36]. This process includes three distinct phases: the elimination phase, where the immune system can effectively recognize and kill malignant cells; the equilibrium phase, in which tumor cells persist, but their growth is restricted by the immune system in a clinical phase of “cancer dormancy”; and the escape phase, where residual cancer cells escape immune control and regrow [37]. The tumor dormancy concept implies that surviving cancer cells remain silent over a long period before regrowing and causing relapses. This concept is supported by the presence of quiescent disseminated tumor cells in animal models and breast cancer patients [38,39]. Besides immunological dormancy, in which T-cells maintain cancer cells in check, two other mechanisms of dormancy have been proposed: cellular dormancy, occurring when tumor cells undergo cell cycle arrest, and angiogenic dormancy, due to insufficient neovascularization [40,41].

The role of type I interferon (type I IFN) signaling in inducing an immune response against tumor cells, cancer immunoediting, and enhancing the efficacy of chemotherapy has been previously studied and reported [42,43,44,45,46,47,48,49,50]. We recently showed that after in vitro exposure to chemotherapy of 4T1 cells, when injected into mice surviving cells induced a strong immune response to produce immunological dormancy in vivo, which was dependent on the active type I IFN signaling pathway [51].

Mathematical modeling of tumor growth and immune system interaction, as a theoretical tool, is advantageous for describing main biological events and developing new treatment modalities, including immunotherapy [52,53,54,55]. Eftimie et al. reviewed non-spatial models, which considered different mathematical equations for components of the tumor-immune complex [56]. Wilkie reviewed the literature of interaction models in the context of cancer dormancy [57]. Wilkie and Hahnfeldt mathematically investigated the evasion of the immune-induced dormant tumor by decaying of immune predation strength and immune recruitment. They also analyzed the immunoediting concept by considering two immune-sensitive and immune resistant subpopulations [58,59]. Page and Uhr presented a model of dormancy induced by antibody production against murine BCL1 lymphoma and investigated the efficacy of prophylactic antibody vaccination. They modeled a tumor consisting of a proliferating and a quiescent subpopulation and their interactions with the antibody and with each other [60]. De Pillis et al. developed a mathematical model to replicate the experimental study of NKG2D ligand-transduced tumor cells, which boosts NK and CD8^+^ T cells activation, stimulates protective immunity as a vaccination, and causes tumor regression [61,62]. These theoretical models have proved that immune components are crucial players in some clinical observations, including tumor dormancy.

This study aims to obtain an evolutionary dynamic model of immunological dormancy, capable of describing the results reported in our previous publication [51], by ordinary differential equations (ODEs). This model introduces two proliferative and quiescent tumor subpopulations and investigates different vaccination scenarios for cancer control.

## 2. Methods

### 2.1. Previously Published Experimental Results

The 4T1 murine tumor is a clinically relevant TNBC model, widely used in cancer research [63,64]. We previously generated a chemoresistant cell line, MR20, by in vitro exposure of the parental wild type (WT) 4T1 cell line to a maximum tolerated dose (MTD) treatment with the anti-metabolite, methotrexate. Chemoresistant MR20 cells grow slightly slower than parental 4T1 cells in vitro [51]. When 5 × 10^4^ MR20 cells were orthotopically injected into the 4th mammary fat pad (MFP) of 6–8 weeks female immunocompetent (wild type) BALB/c mice, four out of ten mice remained tumor-free during one year of study, while the six remaining developed tumors at much later time points compared to mice injected with WT 4T1 cells. Immune profiling of tumor-free mice indicates a significant change from immunosuppression to anti-tumor immune cell infiltration related to the activation of the type I IFN signaling pathway. We concluded that MR20 cells enter a state of immunological dormancy under the active control of the immune system [51], whereas in immunodeficient or T-lymphocyte depleted mice, MR20 cells formed tumors like WT 4T1 cells. The dormancy escaped MR20 variants A1, A3, and B2 arose spontaneously in vitro after a prolonged period of in vivo dormancy [51].

Further, in our published work, we tested whether MR20 cells elicit a protective immune response. To this end, we injected WT MR20 cells in the MFP 10 days before injecting 4T1 parental cells into the contralateral MFP. Results show a reduction in 4T1 tumor growth by approximately 50% in mice preinjected with MR20 cells ([51]).

### 2.2. Assumptions

This study aims to replicate the previously published experimental results in [51], by making a mathematical model of tumor growth and its interaction with the immune system in the TME, based on the studies of Kuznetsov and Makalkin [65], De Pillis et al. [62], and Shariatpanahi et al. [66].

In the process of developing the model, we considered the following assumptions:

1. Under the circumstances of insufficient nutrient supply and cell-contact inhibition, a self-limited growth model best explains the biological facts behind tumor growth. The Gompertzian growth model fits best with the published experimental results and leads to the theoretical maximal tumor size [67,68].
2. The tumor growth rate represents the proliferation rate minus the natural death rate.
3. The considered immune cells in the model represent the directly cytotoxic cells: innate immune effectors, NK cells, and adaptive immune cells, CD8^+^ T lymphocytes (CTL). They can kill tumor cells in a contact-dependent manner or by releasing cytokines and cytotoxic factors [69,70].
4. NK cells, as a part of the innate immune system, are always present and active in tissues, even in the absence of tumor cells; there is a constant source of cytotoxic NK cells.
5. Both NK and CD8^+^ T cells can be inactivated by tumor cells.
6. As the number of dormant cells will remain at about 2000 cells, the dormant tumor might not have enough time to find necessary mutations to escape. For simplicity, the model assumes intra-tumoral heterogeneity as a biological element behind the eventual relapse of the MR20 tumor. In this case, we consider two different phenotypes: a quiescent (MR20) and a proliferative population (4T1).
7. The model does not consider the spatiotemporal heterogeneity of tumor and immune cells in TME.

### 2.3. Mathematical Model

A mathematical model was constructed using an ODEs system. The model includes four different cell populations as variables: proliferative cancer cells (*C_p_*), quiescent cancer cells (*C_q_*), and two effector immune cell populations CD8^+^ T cells (*T*) and NK cells (*N*).

The equations of the model express the change of each population by measuring the number of cells and are solved simultaneously. Equations (1) and (2) describe the evolution of proliferative and quiescent tumor cells, respectively.

(1) $$\frac{dC_{p}}{dt}\;=\;a_{1}C_{p}\ln(\frac{Cmax}{C_{p}})\;-b_{1}NC_{p}\;-\eta_{1}TC_{p},$$

(2) $$\frac{dC_{q}}{dt}\;=\;a_{2}C_{q}\ln(\frac{Cmax}{C_{q}})\;-b_{2}NC_{q}-\eta_{2}TC_{q},$$

The first terms, $aC\;\ln(\frac{Cmax}{C})\;$, indicate a net growth of the cancer population, which follow the Gompertzian growth law. The second and third terms, −bNC and –ηTC, represent the cytotoxicity of NK cells and CD8^+^ T cells on the cancer cells, respectively, by the mass action law. The value of tumor growth rate, a, and the killing rate of tumor cells by immune cells, b and η, differ between these two equations. The parameter, Cmax, is the maximum tumor size.

The dynamic of immune effector cell populations, NK and CD8^+^ T cells, are expressed by Equations (3) and (4), respectively.

(3) $$\frac{dN}{dt}\;=\;s+N(g(\frac{C^{2}}{h+C^{2}}))\;-pCN-fN$$

(4) $$\frac{dT}{dt}\;=\;rNC+T(j(\frac{C^{2}}{k+C^{2}}))\;-uCT-mT$$

$C=\;C_{P}+\;C_{q}$,

The first term in Equation (3), s, is a constant source of NK cells in the tumor microenvironment, and the first one in Equation (4), rNC, indicates that CTLs could be stimulated by the interaction of NK and tumor cells through the release of tumor antigens. The second terms of Equations (3) and (4), $N(g(\frac{C^{2}}{h+C^{2}}))$ and $T(j(\frac{C^{2}}{k+C^{2}}))$ represent a Michaelis–Menten kinetic for recruitment of immune cells; a saturation effect of a limited immune response in the presence of the cancer cells. It means the immune system induction rate increases as the tumor grows until the tumor size reaches a specific value, after which the immune system induction is constant. Here, g and j are the maximum NK and CD8^+^ T cell recruitment rate by the tumor, and h and k are the steepness coefficients of their recruitment curves. The third terms, −pCN and −uCT, show the mass action law of immune cells inactivation after interaction with tumor cells by rates of p and u, and the fourth terms, −fN and −mT, are natural decay terms of immune cells by rates of f and m.

### 2.4. Initial Conditions and Parameters

In the previously reported experimental model, we injected 5 × 10^4^ proliferative (4T1), or quiescent (MR20) cells orthotopically into the mice [51]. The initial number of cytotoxic immune cells, 2.5 × 10^5^ and 5.2 × 10^5^, were estimated based on the differences between the percentage of NK and CD8^+^ T cells in MR20 and saline-injected mice on day 7, respectively.

The tumor growth rates, $a_{1}$ and $a_{2}$, and maximum tumor size, Cmax, were estimated by the growth curve of tumor cells in immunocompromised mice from [71] and [51], respectively. The death rate of quiescent tumor cells by NK cell cytotoxicity, $b_{2}$, was found by fitting the simulations to the growth curve of MR20 tumors in nude mice (Figure S1) [51]. The immunosuppressive tissue microenvironment by the proliferative cells can impair the cytotoxic function of NK cells [69,72,73]. Accordingly, the parameter of NK cell cytotoxicity on the proliferative tumor, $b_{1}$, was considered to be $\alpha b_{2}$; where the assumed coefficient α<1 is the proportion coefficient of NK cells cytotoxicity on proliferative tumor cells compared to quiescent cells. In addition, the rate of CD8^+^ T cells stimulation as a result of proliferative tumor cells killed by NK cells, $r_{1}$, was also assumed to be proportional to $r_{2}$ with the same coefficient α ($r_{1}=\alpha r_{2}$). We estimated the coefficient value by fitting the simulations to our previous experimental data. The death rate of tumor cells by CD8^+^ T cells, η, was estimated from the growth curve of tumor cells in immunocompetent mice [51].

Some of the parameters of cytotoxic immune cell’s equations were obtained from the literature, while others were found by fitting the simulation results to the experimental data from Ref. [51]. The results of fitting indicate that the value of parameters g and j are not different for MR20 tumors relative to 4T1, while the parameters b and η are much larger. The fitting was performed by the least-square distance using an optimization algorithm in MATLAB. Table 1 includes all the parameters, their descriptions, and references.

Since the published data are presented as the tumor volume, the number of cells was calculated based on the unpublished analysis (Table S1).

In order to find the sensitivity of the simulation results to the parameters: initial NK cell number, initial CD8^+^ T cell number, b, η, p, and u, a series of simulations were performed. The parameters were assumed to be normal random variables with the standard deviation value of 25% of the mean value. This standard deviation value was chosen by considering the typical relative errors in the experimental data (Figures S2 and S3).

### 2.5. Simulations

The computational simulations of the model were performed by the Runge-Kutta method, in which each step is determined by the previous step, in the MATLAB R2018b software. Whenever the number of cells goes below one, the relevant population was assumed to be eliminated in the simulation.

Simulation series 1: In these computational experiments, we simulated the proliferative tumor growth by solving Equations (1), (3) and (4) in immunocompetent mice.

Simulation series 2: For the simulation of the quiescent tumors, a single population model was simulated in immunocompetent mice by solving the Equations (2)–(4) simultaneously.

Simulation series 3: These series consider a heterogeneous tumor. Two tumor subpopulations compete for nutrients and space in the TME in an evolutionary dynamic model by modifying the first terms of Equations (1) and (2) and placing the sum of the two populations as the carrying capacity term. The third simulation series were accomplished by considering four populations in the TME: proliferative tumor cells (*C_p_*), quiescent tumor cells (*C_q_*), natural killer cells (*N*), and CD8^+^ T cells (*T*).

In these simulations, the Statements (5)–(9) were used to adjust $a_{1}$, $b_{1}$, $\eta_{1}$, $r_{1}$, and $u_{1}$ parameters by averaging over the relevant parameter of the two populations:

(5) $$a_{1h}\;=\;\left(a_{1}\times C_{p}\right)+\left(a_{2}\times C_{q}\right)/C_{p}+C_{q},$$

(6) $$b_{1h}\;=\;\left(b_{1}\times C_{p}\right)+\left(b_{2}\times C_{q}\right)/C_{p}+C_{q},$$

(7) $$\eta_{1h}\;=\;\left(\eta_{1}\times C_{p}\right)+\left(\eta_{2}\times C_{q}\right)/C_{p}+C_{q},$$

(8) $$r_{1h}\;=\;\left(r_{1}\times C_{p}\right)+\left(r_{2}\times C_{q}\right)/C_{p}+C_{q},$$

(9) $$u_{1h}\;=\;\left(u_{1}\times C_{p}\right)+\left(u_{2}\times C_{q}\right)/C_{p}+C_{q},$$

For heterogeneous tumors, $a_{1h}$, $b_{1h}$, $\eta_{1h}$, $r_{1h}$, and $u_{1h}$ were utilized to replicate a new tumor microenvironment. The biological elements behind this choice are related to the increased concentration of cytokines such as IL-15, IL-7, IL-2, CXCL9, CXCL10, CXCL11, and type I IFNs, which are known activators of the immune effector cells, by the presence of quiescent cells in the TME [75,76].

Simulation series 4: A preconditioning effect of MR20 tumor on the 4T1 tumor growth was previously studied experimentally [51]. Using these data, the most effective vaccination methods were investigated by this series of simulations. To look at the population dynamics of the proliferative tumor challenge after a vaccination (“protective vaccine”), we tested whether injecting dormant cells 20 days before implantation of 4T1 cells could help to get optimum results due to a delay in the immune response to the tumor cell vaccine. Conversely, in a therapeutic setting (“therapeutic vaccine”), quiescent tumor cells should be injected soon (within a week) after tumor induction to be able to control the inoculated proliferative tumor.

## 3. Results

### 3.1. Simulations of Proliferative Tumor Growth (Series 1)

**Figure 1:**
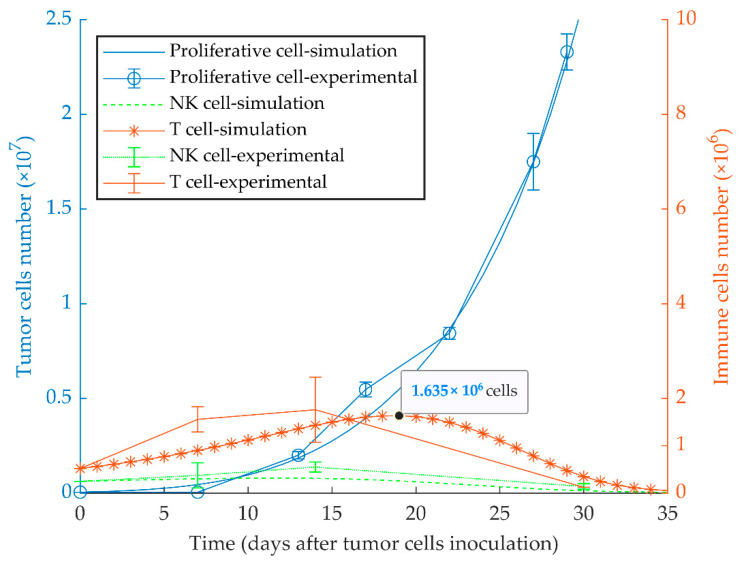
Mathematical simulation of proliferative tumor growth. By simulating the injection of 5 × 10^4^ proliferative tumor cells (WT 4T1) in immunocompetent mice, the tumor overcomes the immune system, and grows exponentially to the lethal phase as in the experimental model. The left and right *y*-axes represent the number of tumor cells and immune cells, respectively. Experimental data are from Reference [51] and are represented as mean ± SEM.

**Figure 2:**
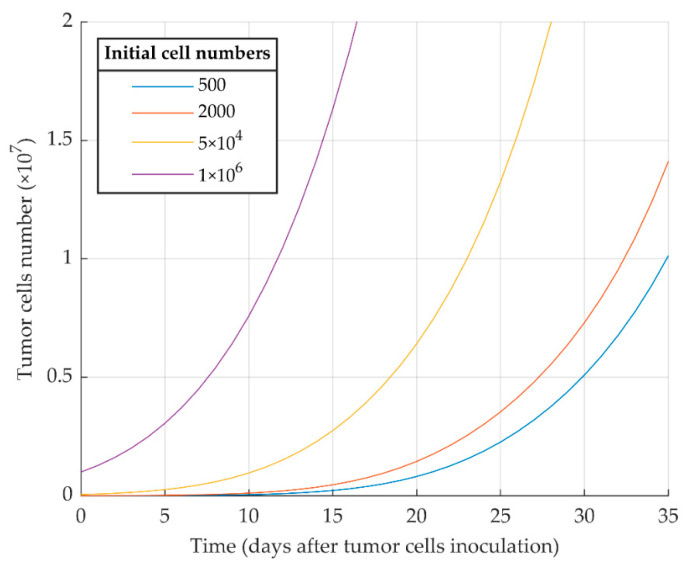
Mathematical simulation of proliferative WT 4T1 tumor growth with different initial cell numbers. The more tumor cells are injected initially, the sooner the tumor exponential growth starts.

The first simulation result indicates that proliferative cancer cells can surpass the immune system by a high growth rate and a low immune-predation rate. As a result, the previously reported experimental results of WT 4T1 growth in immunocompetent mice [51] are well replicated by the model (Figure 1). We also investigated the aggressiveness of our proliferative WT 4T1 tumors by simulating its growth with different initial cell numbers (Figure 2). The results show that the proliferative WT 4T1 tumors, regardless of the initial population, escape the immune system and grow to the lethal phase.

### 3.2. Simulations of Quiescent Tumor Growth (Series 2)

**Figure 3:**
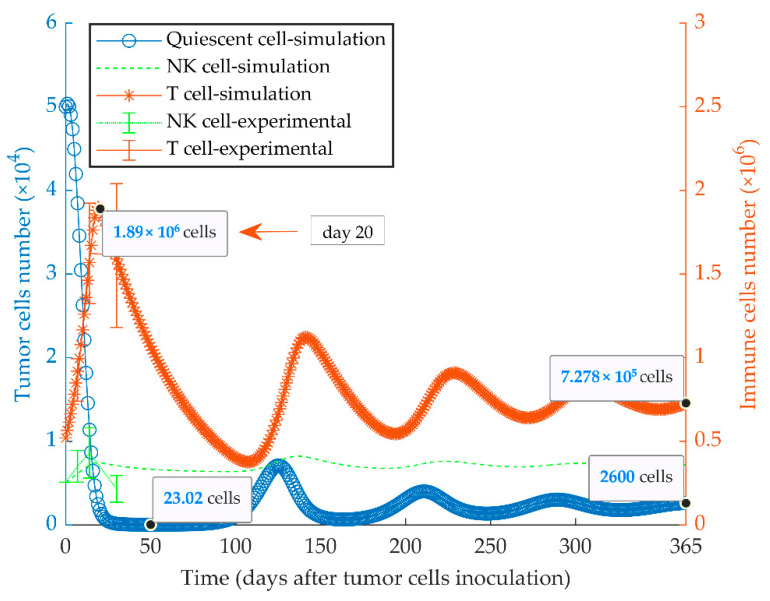
Mathematical simulation of growth of a quiescent tumor. By simulating the injection of 5 × 10^4^ quiescent tumor cells (MR20) in immunocompetent mice, the tumor oscillates around low cell numbers and remains dormant for at least a one-year period. The size of the CD8^+^ T and NK effector cell populations oscillates with a delay lagging tumor cell oscillation. The left and right *y*-axes indicate the number of tumor cells and immune cells, respectively. Experimental data are from Reference [51] and are represented as mean ± SEM.

The immunologically dormant state of the quiescent tumor (MR20) in BALB/c mice considering a uniform population is shown in Figure 3. In these simulations, a high cancer cell kill rate indicates the increased anti-tumor response by CD8^+^ T and NK cells. The effector immune cells can modify cancer growth dynamics to reach a steady-state equilibrium among the populations of tumor cells, NK and CD8^+^ T cells, by an oscillatory pattern. The experimental data of MR20 tumor growth in nu/nu and NSG mice were used to estimate the cytotoxic potency of NK cells and CTLs on the quiescent cancer cells, respectively (Figure S1). Results from the model fully recapitulated the previously obtained experimental results [51].

### 3.3. Simulations of Heterogenenous Tumor Growth (Series 3)

**Figure 4:**
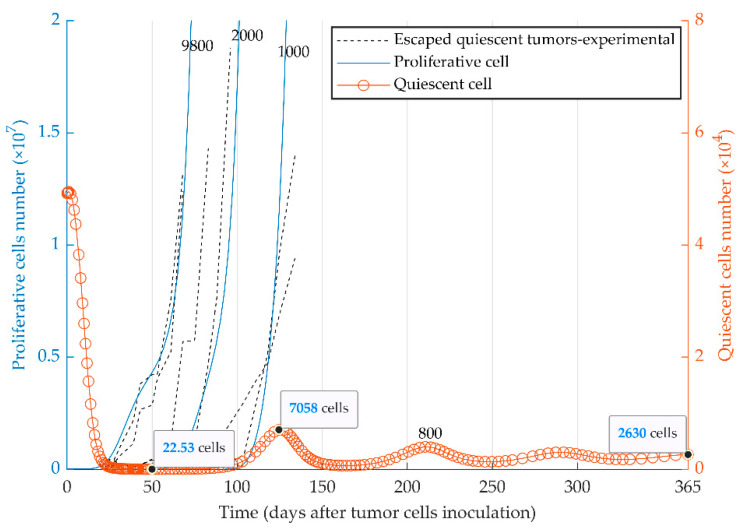
Mathematical simulation of the fate of heterogeneous tumors. Two subpopulations, proliferative (blue curve) and quiescent (red curve), are present within the 5 × 10^4^ injected tumor cells. The fate of the tumor is represented by the simulation with four different initial numbers of proliferative cells: 8 × 10^2^, 1 × 10^3^, 2 × 10^3^, 9.8 × 10^3^. Experimental graphs for escaped tumors [51] are also replicated in the figure. Due to different scales, the left and right *y*-axes present the number of proliferative and quiescent tumor cells, respectively. The elimination events, where the number of tumor cells becomes smaller than one during the simulation, are not shown due to the large scale of the diagram.

The evolutionary dynamics of the two cancer phenotypes (i.e., quiescent and proliferative populations) were considered in the third series of simulations. Dormancy, elimination, and growth states of tumor cells can be simulated by the injection of a heterogeneous tumor cell population into immune-competent mice. The initial number of proliferative cells determines the fate of the heterogeneous tumor (Figure 4) in vivo. The model reproduced the escape events of quiescent tumors at different time points (Figure 4) as observed experimentally with MR20 cells in vivo ([51]). The quiescent tumor cells remain dormant (red curve for the quiescent cells) when a very small number of proliferative cells were initially injected (e.g., 800 cells). In contrast, tumors with the initially higher number of proliferative cells escape the immune system (blue curves for the proliferative cells) from the time of injection to around day 130.

**Figure 5:**
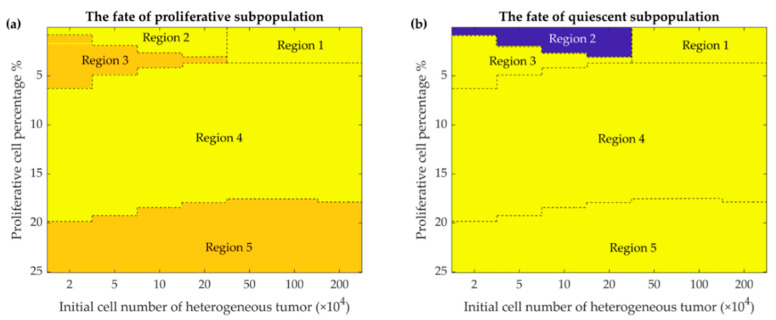
Phase maps of the fate of the two subpopulations present in the heterogeneous tumor after 365 days. The fate of the proliferative (**a**) and quiescent (**b**) subpopulations depends on the percentage of initially present proliferative cells (up to 25 percent are shown) in the different initial tumor cell populations (from 2 × 10^4^ to 2 × 10^6^ cells). Color-coded events: blue, dormancy; yellow, escape; orange, elimination events (See discussion for further details).

Mathematical simulation of intra-tumoral heterogeneity dynamics depicts the fate of two subpopulations based on the initial tumor size and the percentage of initial proliferative cells (Figure 5). The three final states of the heterogeneous tumor and its two clades are shown by different regions in the phase maps (Figure 5) and summarized in Table 2. These results show that the heterogeneous model escapes in the presence of a high proliferative cell percentage, stays in a dormant state with a small number of proliferative cells during one year of simulation, or is fully eliminated with intermediate number of proliferative cells.

### 3.4. Simulations of Vaccination Scenarios (Series 4)

**Figure 6:**
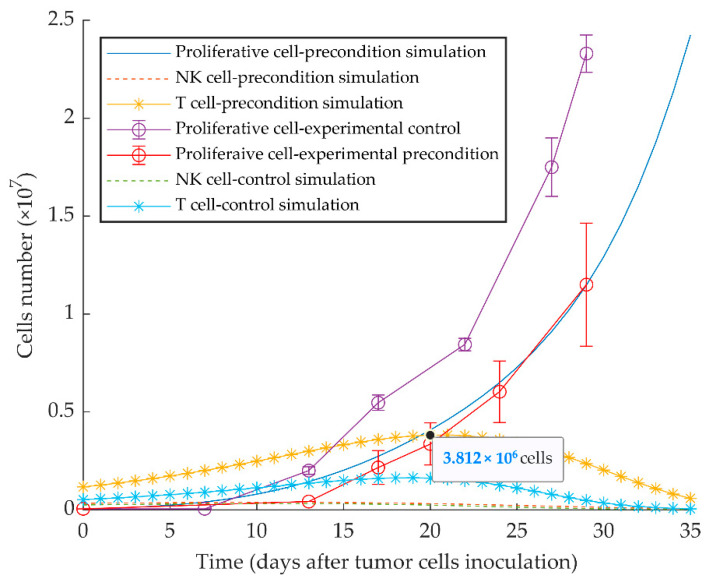
Mathematical simulation of aggressive tumor growth delay by preconditioning of mice with dormant tumor cells. By simulating the injection of 5 × 10^4^ quiescent tumor cells 10 days before injecting the same number of proliferative tumor cells on day 0, the simulation shows increased stimulation of CD8^+^ T cells and subsequent delayed tumor growth. Experimental data are from Reference [51] and are represented as mean ± SEM.

The model appropriately replicates the vaccination effect of injecting quiescent tumor cells 10 days before the injection of the same number of proliferative cells, as experimentally reported in [51]. The delayed growth of proliferative tumor cells is the result of the increased protective immune response elicited by the preconditioning with quiescent tumor cells (Figure 6).

**Figure 7:**
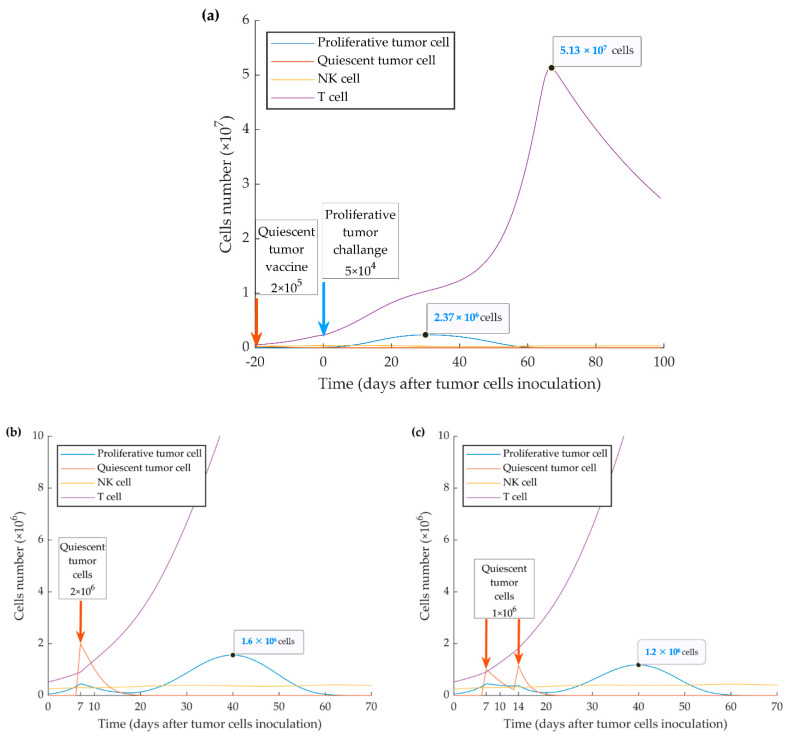
Mathematical simulation of a vaccination protocol. By injecting simulation of higher numbers (i.e., >2 × 10^5^ cells) of quiescent cells (**a**) 20 days before implantation of wild type (WT) tumor cells, tumor growth can be suppressed. Inoculation of one-time 2 × 10^6^ quiescent tumor cells 7 days after implantation of WT tumor cells (**b**) or twice 1 × 10^6^ quiescent tumor cells 7 and 14 days after implantation of WT tumor cells (**c**) resulted in further or complete suppression of tumor growth, respectively.

As shown in Figure 3, the time of immune activation peak is around day 20 after injection of quiescent tumor cells. This is consistent with the fact that mounting an immune response and maturation of CD8^+^ T cells in response to tumor cell injection require time. When we simulated the injection of the quiescent tumor cells at 20 days before the implantation of aggressive tumor cells, we observed that the evasion of the tumor could be fully suppressed. However, the simulation also shows that this outcome only occurs when a four-fold higher number of quiescent cells (2 × 10^5^) is injected (ipsilaterally or contralaterally) (Figure 7a). The model also investigated the treatment vaccination possibility to prevent the escape of the subsequent injecting proliferative cells by increasing the number of active cytotoxic T lymphocytes. The simulation results display two successful treatment schedules by ipsilateral injecting 2 × 10^6^ quiescent tumor cells at 7 days or twice 1 × 10^6^ at days 7 and 14 after WT tumor cell injection (Figure 7b,c).

## 4. Discussion

Published literature reported the activation of the type I IFN pathway through ICD induction by some chemotherapeutic agents, such as anthracyclines, cyclophosphamide, and oxaliplatin [27,28,51]. Some studies indicate a transient activation of type I IFN signaling after one cycle of chemotherapy, and the active pathway suppresses metastasis formation whilst not affecting the growth kinetics of the primary tumor [45,49]. We recently demonstrated that the type I IFN pathway is a promoter of anti-tumor immune responses in breast cancer and, in particular, is an inducer of immunological dormancy. Our previously published experimental results [51] showed that the immune-stimulatory effect of sustained type I IFN signaling promotes permanent primary tumor dormancy in four out of 10 immunocompetent mice, one year after in vivo inoculation. Specifically, the upregulation of IRF-7, a regulator of the pathway, resulted in the expansion of dendritic cells and T lymphocytes and the suppression of the mobilization of myeloid-derived suppressive cells (MDSCs) [51]. Interestingly, compared to WT (parental) 4T1 cells, MR20 cells, expressed higher levels of MHC-I complexes restricting antigen presentation to CD8 T cells, consistent with increased immunogenicity of dormant cells (unpublished observation).

Based on these data, we generated a functional model considering the net effect of many cytokines based on their role in immune cell communications and functions. Such a minimally parameterized model can be more easily tested and validated and is more flexible in representing the fundamental dynamics behind the tumor-immune system interaction. In this study, we confirm the dormant state of chemotherapy-induced quiescent cells by the mathematical model (Figure 3). The experimental data translate to the higher immune cytotoxicity rate, $\eta_{2}$, by the high expression of MHC-I, induction of NK and CD8^+^ T cells and the reduction of MDSCs. The lower growth rate, $a_{2}$, could be partly due to the apoptotic activity of type I IFNs after in vitro chemotherapy. However, the inactivation of type I IFN pathway results in the decrease of the effective immune response against MR20 tumors and the consequent tumor growth. Experimentally, the escaped tumors are associated with a significant decrease in the expression of IFN-β [51]. Our experimental data already indicated that some patients have a more effective IFN-β response than others in response to chemotherapy, and those may be the patients that respond with a stronger immune response and prolonged dormancy [51]. Understanding the regulation of type I IFN expression in cancer cells will be an important subject of future investigations.

In our original publication [51], we showed that in MR20 injected mice, the number of B cells increases in parallel to T cells and NK cells, suggesting a possible involvement of B cells and humoral response in dormancy. In humans, anti-HER-2 antibodies are effective in inducing cell-cycle arrest and cancer cell death involving, at least in part, antibody-dependent cytotoxicity, and NK cell activation [77,78,79,80]. In our mathematical model, however, we did not consider B cells and the humoral response because we did not functionally demonstrated their contributions. Although not considering the effect of humoral immunity, our model shows that there is probably an effective humoral response. The estimated antigenicity values for 4T1 and MR20 cells (j) are found to be almost the same in the parameter calibration process. This is in contrast to the experimental results of MHC-I expression (unpublished observation). The difference is likely due to the considerable recruitment of B cells and their consequent effective immune response. This effect is also observable in the large difference between the cytotoxicity values $\eta_{1}$ and $\eta_{2}$. It is, however, an issue to reconsider in future works, given the fact that experimental evidence suggests that B cells and the humoral response may have both tumor-suppressive [81,82] and tumor-promoting [83,84] effects. 

The exact mechanism of how quiescent (MR20) tumors escape immune control is unknown at this time. We proposed and mathematically modeled a mechanism based on an intra-tumor heterogeneity and assumed a competition between the two different phenotypes, one with an inactive type I IFN pathway and the other with upregulated type I IFN signaling. The evolutionary dynamics are formulated considering the direct competition of the two tumor cell types due to the limited resources, in addition to the indirect competition caused by the immune response. While the direct competition mostly plays a role when the tumor escapes, the indirect competition becomes significant during dormancy. Further, the indirect competition establishes a promising vaccination hypothesis. It should be noted that although this is a relatively simple model, the integration of the immune-editing related patterns and the evolutionary dynamics in the model generates complex behaviors.

In regards to the immune stimulation delay as a biological and mathematical nature of the model, a large number of the proliferative cells break the immune control and grow to the lethal phase while the quiescent cells are eradicated from the TME. On the other hand, the intermediate number of the initially proliferative cells results in the extinction of both tumor subpopulations. This elimination originates from the oscillation of the predator (immune system) prey (tumor cells) model [57,85,86,87]. When the amplitude of the oscillation is too large, the number of the tumor cells reaches values less than one, and the tumor would be eradicated (Figure 4). Finally, with a low percentage of the proliferative cells, when the tumor cannot escape the immune system, nor it is eliminated, it starts to oscillate until the oscillation damps to an equilibrium called dormancy (Figure 3 and Figure 4) [65].

Figure 5 shows the fate of the tumor for different conditions of the initial number of tumor cells and their combinations. As the figure shows, when the proportion of proliferative cells is very small (and the tumor is mostly quiescent), two fates can be imagined for the tumor: for a relatively larger number of tumor cells, large amplitude oscillations would eliminate the tumor. This is due to the fact that the initial number of the tumor cells, hundreds of thousands, is much larger than the equilibrium (few thousand quiescent cells) (Region 1, Figure 5a,b). Conversely, when the initial number of the tumor cells is relatively smaller, a quiescent dormant tumor would survive through the smaller oscillation amplitudes (Region 2, Figure 5b). As the number of initial proliferative cells increases, for smaller heterogenous tumor (Region 3, Figure 5a,b), the proliferative tumor finds a chance to escape; meanwhile, the quiescent cells would be eliminated due to the competition with the aggressive proliferative cells. As the number of proliferative cells further increases (Region 4, Figure 5a,b), the rapid growth of the proliferative tumor results in a large immune response, and large amplitude oscillations, in turn, eliminate both subpopulations of the tumor. Finally, for different initial tumor sizes and larger proportions of proliferative cells, the tumor escapes as expected for typical 4T1 tumors; the quiescent subpopulation of the tumor would be eliminated by the proliferative subpopulation (Region 5, Figure 5a,b).

In line with other studies [68], we also show that the WT 4T1 cell line generates an aggressive tumor, which grows in immunocompetent mice with different kinetics depending on the number of initially injected cells (Figure 2). It seems that the tumor cell kill rate by CTLs, $\eta_{1}$, is low because of the suppression of the immune system by MDSCs, which are more recruited by proliferative cells [45,51].

Due to the effect of activated type I IFN pathway in the quiescent (MR20) cell line, we also show by the simulation that a vaccination effect can be achieved through the injection of quiescent cells 10 days before the inoculation of proliferative cells (Figure 6). By simulating injection of 2 × 10^5^ or more quiescent cells 20 days before the implanted proliferative tumor, the latter can be brought under the control of the immune system (Figure 7a). Further, two treatment schedules were successfully simulated by injecting 2 × 10^6^ quiescent tumor cells 7 days, or by injecting twice 1 × 10^6^ quiescent tumor cells 7 and 14 days after WT tumor implantation (Figure 7b,c). These results indicate that preventive and therapeutic vaccination strategies using dormant cancer cells may keep disseminated aggressive tumor cells under control by increasing the number of activated cytotoxic CD8^+^ T cells. Diefenbach et al. induced high-level expression of activating NKG2D ligands in melanoma, lymphoma, and thymoma cell lines by a retrovirus expression system. The study concluded the activation of NK cells and CD8^+^ T cells, with tumor transduced with NKG2D ligand, lowers tumor growth incidence, and stimulates protective immunity to tumor challenge with ligand-negative tumor cells [88]. Ravindranathan et al. confirmed the failure of the 4T1 cell vaccine because of highly splenic and tumor site accumulation of MDSCs and immune impairment by increased expression of granulocyte colony-stimulating factors. By the increased immunogenicity and protective effect of the 4T1.G-CSF^−^ vaccine, the results implied that tumor-derived factors are more important for establishing an immunogenic breast cancer vaccine than surface phenotype [89]. Further, therapy with cytokines, such as IL-2 gene therapy in a metastatic breast cancer model, can induce anti-tumor immunity that represses the growth of primary tumors or subsequent challenges [90,91,92]. To these points, autologous tumor cell vaccine with the immunogenic quiescent tumor cells, possibly in combination with IFN-β, may help to present the entire antigen to the immune system and suppress negative immune regulatory mechanisms.

Recently, some tumor-cell based vaccines were introduced and are under active clinical trials. Triplex is an allogenic preventive vaccine and is made by HER-2 transgenic mammary carcinoma cells, which are transduced with class I major histocompatibility complex (MHC-I) and IL-12 genes [93]. Another allogenic vaccine is SV-BR-1-GM (BriaVax), consisting of irradiated HER-2/neu positive breast cancer cells transduced with granulocyte/macrophage-colony stimulating factor (GM-CSF), which was introduced to treat advanced breast cancer cases. Specifically, 15–25 × 10^6^ cells are inoculated intra-dermally 2–3 days after intravenous injection of cyclophosphamide, then IFN-α is injected after 2–4 days into the sites of implantation [94]. Thus, MR20 tumor with immune-stimulatory hallmarks, including GM-CSF overexpression [51], and type I IFN signature is a convenient choice of vaccination, which is suggested by our model.

## 5. Conclusions

Mathematical modeling of tumor-immune system interaction was chosen as an approach to decipher the complex phenomenon of immunological dormancy and to generate experimentally verifiable predictions. Immunological tumor dormancy is traditionally considered as a labile state: it may continue during the patient’s lifespan resulting in quiescent cancer ending in cure if the tumor is completely eliminated, or it can lead to recurrence if dormancy is broken. Based on our previously published results, we propose here an evolutionary dynamic mathematical model that reproduces these experimental events and provides evidence for effective preventive and therapeutic vaccination strategies. This model not only faithfully reproduced the experimental data, but it also generated a novel testable hypothesis suggesting that dormant cancer cells may be considered as a vaccine to blunt the progression of aggressive breast cancer and prevent metastasis formation. Eventually, enhancing the cytotoxic immune response against tumors to eradicate or keep breast cancer as a chronic, quiescent, or stable disease may be achieved by combining immunotherapy, cytokines, or cancer cell vaccines, to the chemotherapy.

## Figures and Tables

**Table 1:** Estimated Parameter Values.

Parameters	Units	Estimated Value	Description	Source
$a_{1}$	Day^−1^	0.38 × 10^−1^	Proliferative (WT 4T1) tumor growth rate	[71]
$a_{2}$	Day^−1^	0.174 × 10^−1^	Quiescent (MR20) tumor growth rate	estimated from [51]
$C_{max}$	Cell	10^9^	Maximum tumor size	estimated
α	Dimensionless	0.3	Proportion coefficient of NK cell cytotoxicity on tumor cells	estimated
$b_{1}$	Cell^−1^ Day^−1^	$\alpha \left(b_{2}\right)$= 1.56 × 10^−8^	Proliferative tumor cells kill rate by NK cells	estimated from [51]
$b_{2}$	Cell^−1^ Day^−1^	5.2 × 10^−8^	Quiescent tumor cells kill rate by NK cells	estimated from [51]
$\eta_{1}$	Cell^−1^ Day^−1^	0.21 × 10^−7^	Proliferative tumor cells kill rate by CD8^+^ T cells	estimated from [51]
$\eta_{2}$	Cell^−1^ Day^−1^	2.8 × 10^−7^	Quiescent tumor cells kill rate by CD8^+^ T cells	estimated from [51]
s	Cell Day^−1^	1.3 × 10^4^	Constant source of NK cells.	[62,65]
$r_{1}$	Cell^−1^ Day^−1^	$\alpha \left(r_{2}\right)$= 0.36 × 10^−7^	CD8^+^ T cells stimulation coefficient due to proliferative tumor-NK cells interaction	estimated
$r_{2}$	Cell^−1^ Day^−1^	1.2 × 10^−7^	CD8^+^ T cells stimulation coefficient due to quiescent tumor-NK cells interaction	[62,66] estimated
g	Day^−1^	2.5 × 10^−2^	Maximum NK cell recruitment rate by tumor cells	[62,66]
h	Cell^2^	2.02 × 10^7^	Steepness coefficient of the NK cell recruitment curve	[62,66]
f	Day^−1^	4.12 × 10^−2^	Death rate of NK cells	[62,66,74]
p	Cell^−1^ Day^−1^	1.8 × 10^−8^	NK cell inactivation rate by tumor cells	estimated from [51]
j	Day^−1^	10 × 10^−2^	Maximum CD8^+^ T-cell recruitment rate by tumor cells	[66]
k	Cell^2^	2.02 × 10^7^	Steepness coefficient of the CD8^+^ T-cell recruitment curve	[62,65,66]
m	Day^−1^	2 × 10^−2^	Death rate of CD8^+^ T cells	[62,66]
$u_{1}$	Cell^−1^ Day^−1^	2.1 × 10^−8^	CD8^+^ T-cell inactivation rate by proliferative tumor cells	estimated from [51]
$u_{2}$	Cell^−1^ Day^−1^	1.7 × 10^−12^	CD8^+^ T-cell inactivation rate by quiescent tumor cells	estimated from [51]

**Table 2:** The Cellular Fate within the Heterogenous Tumors.

Regions of Phase Map	Quiescent Subpopulation	Proliferative Subpopulation	Heterogeneous Tumor State
Region 1	Eliminated	Eliminated	Eliminated
Region 2	Dormant	Eliminated	Dormant
Region 3	Eliminated	Escaped	Escaped
Region 4	Eliminated	Eliminated	Eliminated
Region 5	Eliminated	Escaped	Escaped

## Data Availability

Data are available upon written request.

## Supplementary Materials

The following are available online at https://www.mdpi.com/2072-6694/13/2/245/s1, Table S1: Measurement of tumor cell number, Figure S1: Experimental and simulated growth curves of MR20 tumors, Figure S2: Sensitivity analysis of proliferative tumor parameters, Figure S3: Sensitivity analysis of quiescent tumor parameters.
