# Supplementary material for: Dormant Tumor Cell Vaccination: A Mathematical Model of Immunological Dormancy in Triple-Negative Breast Cancer
Source: Cancers (Basel). 2021 Jan 11;13(2):245. doi: 10.3390/cancers13020245 (PMC7827392; doi:10.3390/cancers13020245)
Supplement: Supplementary file 1 [file cancers-13-00245-s001.pdf]

cancers

Supplementary Materials: Dormant Tumor Cell Vaccination: A Mathematical Model of Immunological Dormancy in Triple-Negative Breast Cancer

Reza Mehdizadeh, Seyed Peyman Shariatpanahi, Bahram Goliaei, Sanam Peyvandi and Curzio Rüegg

Citation: Mehdizadeh, R.; Shariatpanahi, S.P.; Goliaei, B.; Peyvandi, S.; Rüegg, C. Dormant Tumor Cell Vaccination: A Mathematical Model of Immunological Dormancy in Triple-Negative Breast Cancer. *Cancers* **2021**, *13*, 245. https://doi.org/10.3390/cancers13020245

Received: 22 December 2020
Accepted: 07 January 2021
Published: 12 January 2021

Publisher's Note: MDPI stays neutral with regard to jurisdictional claims in published maps and institutional affiliations.

Copyright: © 2021 by the authors. Submitted for possible open access publication under the terms and conditions of the Creative Commons Attribution (CC BY) license (http://creativecommons.org/licenses/by/4.0/).

Table S1. Measurement of tumor cell number. After injection of 5×10^4 proliferative or quiescent tumor cells mice were sacrificed at day 9, 15 or 30. The number of tumor cells and the percentage of CD45$^+$ leukocytes were measured at each time point. For each time point, three mice were sacrificed.

Days from Injection	Mean Cell Number (4T1) ± SEM (Millions)	% CD45$^+$ Cells	Mean Cell Number (MR20) ± SEM (Millions)	% CD45$^+$ Cells
**9**	11.2 ± 3.17	95	6.7± 0.25	95
**15**	17.7 ± 1.17	78	9± 1.6	95
**30**	33.5± 13.79	60	11.3± 0.88	95

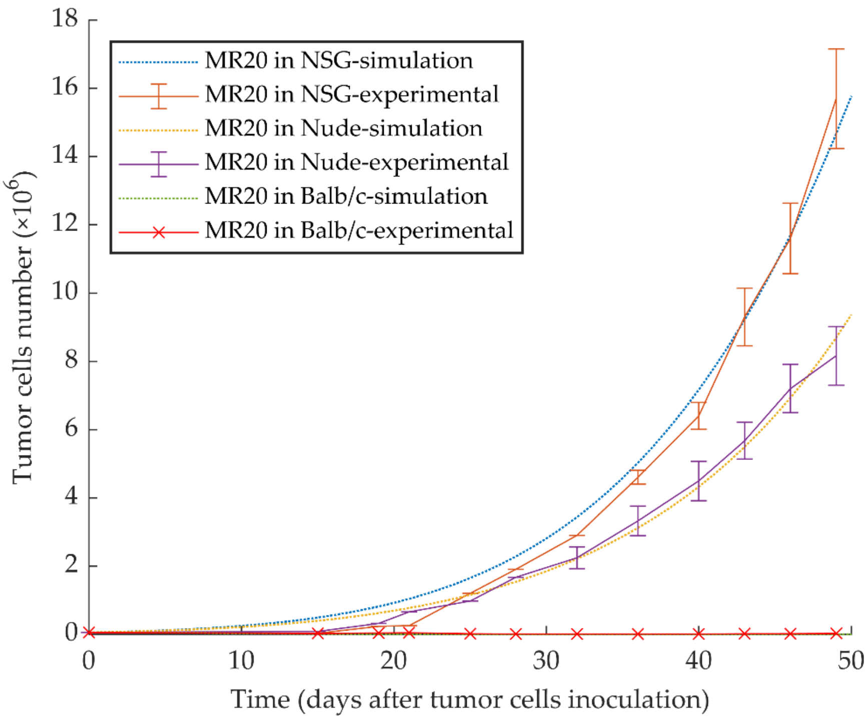

Figure S1. Experimental and simulated growth curves of MR20 tumors. MR20 tumor cells were injected into three different types of mice: wild type BALB/c mice, nu/nu mice and NSG (NOD SCID common gamma 2 KO) mice. In each group, at least five mice were included. Experimental data are represented as mean ± SEM. Experimental data are from Ref. [51].

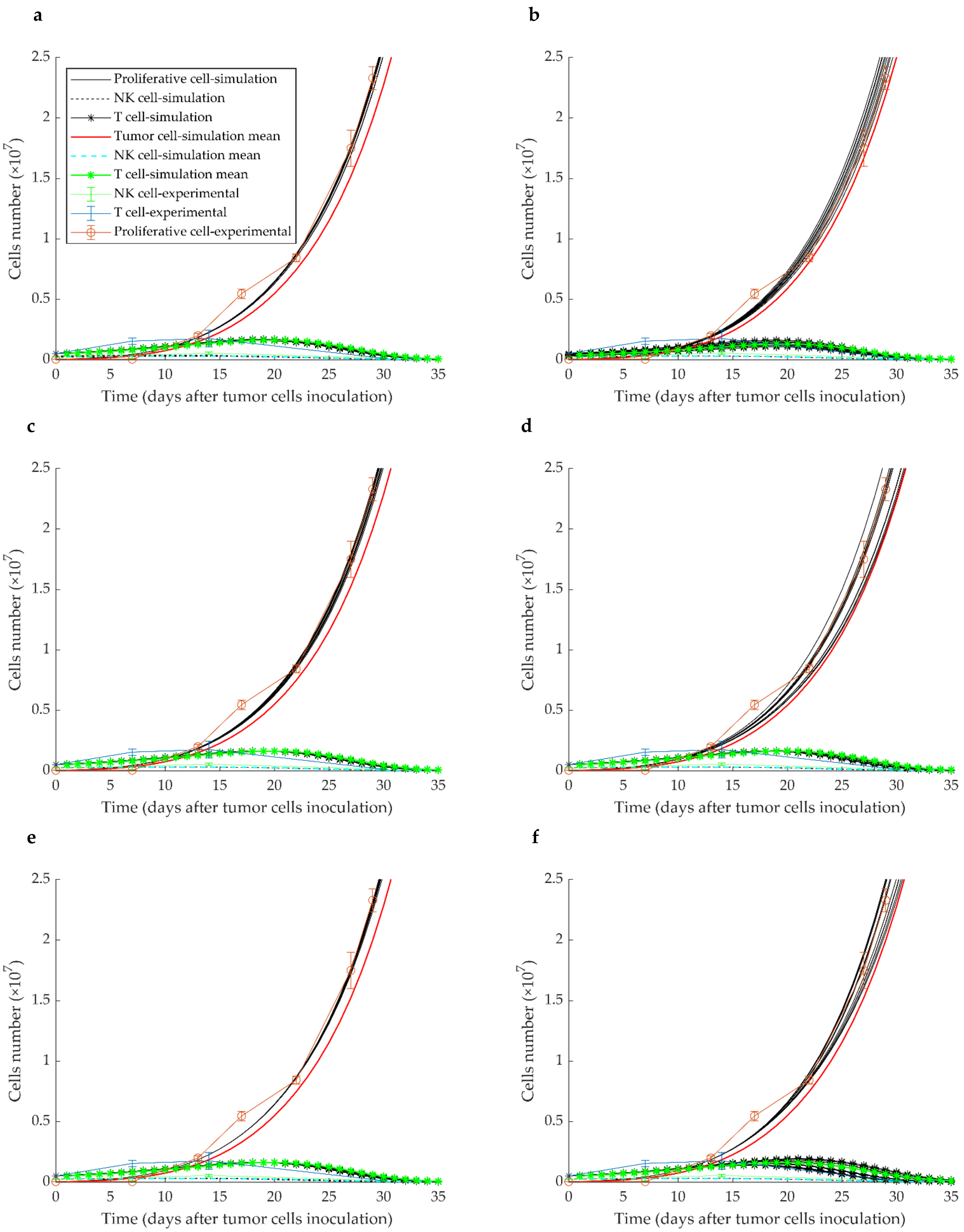

Figure S2. Sensitivity analysis of proliferative tumor parameters. The effector immune system's initial conditions and the equation parameters were randomized by a normal random variable with a standard of deviation (SD) of 25% of the mean value. Each simulation was performed 10 times. The average curve was calculated for each series of curve simulations of

tumor cells, NK cells and T cells. (**a**) initial NK cell number, (**b**) initial CD8+ T cell number, and parameters (**c**) b_1, (**d**) η, (**e**) p, (**f**) u_1 were randomized.

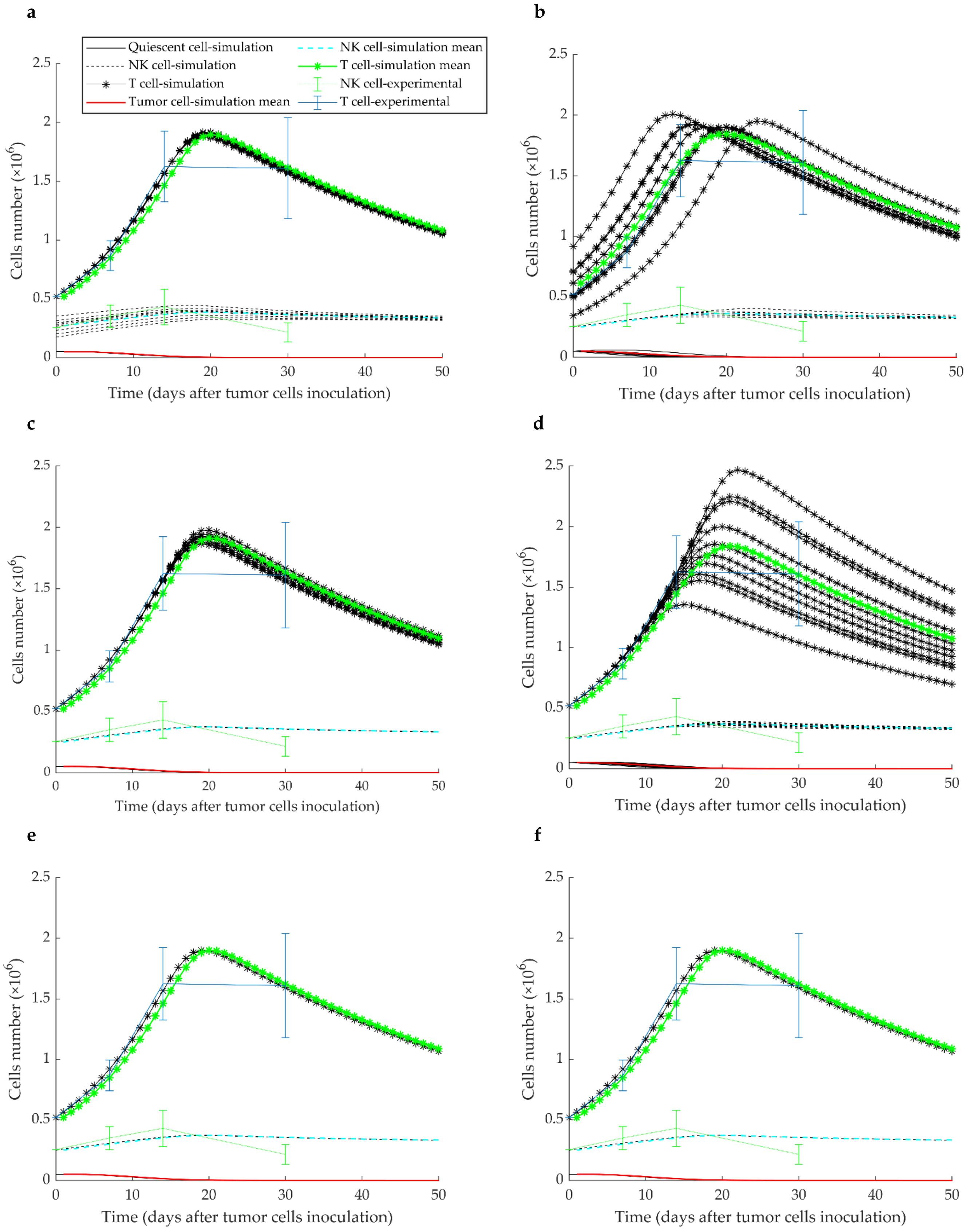

Figure S3. Sensitivity analysis of quiescent tumor parameters. The effector immune system's initial conditions and the equation parameters were randomized by a normal random variable with a standard deviation (SD) of 25% of the mean value. Each simulation was performed 10 times. The average curve was calculated for each series of curve simulations of tumor cells, NK cells and T cells. (**a**) initial NK cell number, (**b**) initial CD8+ T cell number, and parameters. (**c**) b, (**d**) η, (**e**) p, (**f**) u_2 were randomized.
